# Modern Sociology

**Published:** 1900-11-03

**Authors:** 


### Modern Sociology.

### SOME DANGEROUS TRADES.

### I.—File-Cutting.

In 1895 a committee was appointed by the Home Office to inquire into the nature of certain dangerous trades, and to suggest methods by which the risk to the life and health of the workers might be lessened. The committee consisted of Mr. H. J. Tennant, M.P.; his wife, formerly Miss May Abrahams, H.M. Superintending Inspector of Factories; Dr. Thomas Oliver, M.D., F.R.C.P., Physician to the Royal Infirmary, Newcastle-on-Tyne; and Mr. Hamilton P. Smith, retired Commander R.N., H.M. Inspector of Factories. The committee finished its work last year, and has presented the results in three interim reports and a final one. We purpose giving some account of the various trades investigated by the committee.

First we take the report on file-cutting. File-cutting by machinery hardly ranks among dangerous trades, for it is done in modern "shops," where the ventilation and air-space are sufficient, and the "bed," of which we shall speak presently, is of pewter. But in file-cutting by hand the conditions are very different. In the first place, the "shop," as it is called, is frequently some shed or outhouse, attached to a dwelling-house, and, in many cases, in immediate proximity to middens, privies, and cesspools. Ventilation can be obtained only by opening the windows, which are so arranged that the light falls mainly on the operative and his work. It can, therefore, be easily understood that the average worker prefers a close atmosphere to a draught, which would, moreover, blow directly down his throat the dust, a mixture of lead and steel, generated by his work.

The worker sits in front of a stone block, called a "stock." In the centre of this is placed a smaller steel block, slightly raised above the surface of the stock, which is called a "stiddy." On this again a piece of lead, a little larger than the file that is to be cut, is placed; this is the "lead bed." The lead is sufficiently soft to prevent a recoil which would jar the worker when he cuts the teeth of the file by a stroke with a hammer and chisel, and when the work is heavy the file flattens out for itself a groove in the lead, in which it can lie without moving. It is also kept in position by leather straps passed over each end of the file. One end of these straps is kept tightly fixed between the "stiddy" and the "stock," while the other forms a stirrup which the worker braces with his foot, to keep the file from moving. When one face of the file has been cut, it is rubbed with charcoal before it is turned, and when both are cut the file is briskly brushed. When the file is large, and the teeth to be cut on it deep, the work is very heavy. The hammer with which the blow is given to the chisel weighs from 7½ to 9 lbs., and it has been calculated that in a day's work a man may lift 190 tons. The two sides of a 12-inch file require the cutting of 3,800 teeth; and this, with an 8-lb. hammer, would entail the lifting of 13 tons per file.

It is not, however, in the severity of the work that its danger lies. There are, in the first place, the insanitary conditions in which it is carried on. It might seem that the Factories Acts give ample power for the remedying of these. But these file-cutters are very much the position of these independent workmen whom the lauders of times past profess to envy. Some have stocks at home. If the master of the house makes his living by the work, the law can enter and supervise the conditions under which he labours; but the law is powerless when, as happens sometimes, the wife and children do some file-cutting in the intervals of other duties. Even when not prosecuted as a home industry, file-cutting is carried on in a way that makes it difficult to control it. Each "stock" in a shop may be rented separately by the man or woman who works at it, and every occupant of a shop may be working for a different firm. Who, then, is responsible for the condition of the shop? Is it the workers themselves, or the owner of the "shop," or the firm or firms for whom the work is done? At present none of these seem to be accountable.

The main cause of trouble is, however, the lead bed As the hammering presses the file into this, dust is generated, which, mingled with that from the steel filings and charcoal, is inhaled by the workers. The fact that the workers often eat their dinner in the shop, usually without taking the trouble to wash their hands, makes it all the more likely that they will swallow this dust, and thus they suffer much from lead-poisoning. As they do not change their clothes, nor take a proper bath when they go home, their families share in the infection. The dangerous nature of the trade has been frequently noticed by medical men. In 1893 Dr. Sinclair White, Lecturer on Public Health at the Sheffield Medical School, in a lecture delivered to the Sanitary Institute of London, said that there were 4,000 men, besides a large number of women engaged in file-cutting. He had investigated the cases of 100 of these, taking them haphazard as he met them in the shops. Of the 100 seventy-four had a "lead line" in their gums, twenty-eight had had lead colic, and twenty had at some time suffered from paralysis of the wrist and thumb. The average age of these workers was thirty-seven years. Dr. H. H. Littlejohn, who was for six years Medical Officer of Health for Sheffield, says that in his experience it is rare to find a robust file-cutter. Even when they cannot be specifically declared to be suffering from lead-poisoning, they are weak and anæmic, and Dr Littlejohn is of opinion that many deaths are recorded as being due to secondary and dependent diseases which were primarily caused by lead-poisoning. The Registrar-General also declares in a report that "exposure to lead-poisoning is associated with increased liability to disorders of the urinary and nervous systems. It is found that those occupations which show the greatest excess of mortality from plumbism also show the greatest excess of mortality from diseases of the urinary and nervous systems." He points out that among file-makers the deaths from these causes combined is as 316 against 123 among occupied males in general.

The committee think that the conditions of the work might be improved by making the person who gives out work responsible for the place in which it is done; and also by making the occupier or occupiers of a shop provide soap, water, basins and towels for the workers. It is suggested also that the workers should be obliged to wear overalls, which the occupiers of the shop should take care were washed once a week. We think that these suggestions, if enforced, would probably result in the work being removed from these unhealthy sheds to proper factories, which would probably be the best remedy of all.

